# Echo chamber detection and analysis: A topology- and content-based approach in the COVID-19 scenario

**DOI:** 10.1007/s13278-021-00779-3

**Published:** 2021-08-21

**Authors:** Giacomo Villa, Gabriella Pasi, Marco Viviani

**Affiliations:** Information and Knowledge Representation, Retrieval, and Reasoning (IKR3) Lab - Department of Informatics, Systems, and Communication, University of Milano-Bicocca, Milan, Italy

**Keywords:** Echo chambers, Social media, Social network analysis, Community detection, Sentiment analysis, Topic modeling, COVID-19

## Abstract

Social media allow to fulfill perceived social needs such as connecting with friends or other individuals with similar interests into virtual communities; they have also become essential as news sources, microblogging platforms, in particular, in a variety of contexts including that of health. However, due to the homophily property and selective exposure to information, social media have the tendency to create distinct groups of individuals whose ideas are highly polarized around certain topics. In these groups, a.k.a. echo chambers, people only "hear their own voice,” and divergent visions are no longer taken into account. This article focuses on the study of the echo chamber phenomenon in the context of the COVID-19 pandemic, by considering both the relationships connecting individuals and semantic aspects related to the content they share over Twitter. To this aim, we propose an approach based on the application of a community detection strategy to distinct topology- and content-aware representations of the COVID-19 conversation graph. Then, we assess and analyze the controversy and homogeneity among the different polarized groups obtained. The evaluations of the approach are carried out on a dataset of tweets related to COVID-19 collected between January and March 2020.

## Introduction

In the present era, also known as *information age* (Floridi 2014), people are exposed to a significant amount of online content. Social media, in particular, have led to a drastic shift in the size and velocity at which information is communicated, and social media feeds are essential resources for accessing vast volumes of news and other types of informative contents in real time. In these platforms, people have the possibility to be connected and share both *conversational* and *newsworthy* content with friends and/or strangers into virtual communities (Zubiaga et al. 2016), almost without any form of traditional trusted intermediation (Eysenbach 2008; Carminati et al. 2012).

This leads to two main open issues. First of all, to the so-called *information overload* problem (Melinat et al. 2014): faced with such a volume of information, users are often unable to discriminate between *relevant* and *non-relevant* one. Secondly, to the *information disorder* problem (Wardle and Derakhshan 2017): the current online ecosystem is polluted with different types of non-genuine information, the so-called *dis-*, *mis-*, and *mal-information*. Both problems can lead to detrimental consequences for society, even in very sensitive contexts such as the *health-related domain* (Klerings et al. 2015; Waszak et al. 2018). In this context, coming into contact with irrelevant or unverified content can have serious repercussions on public health.

To face the information overload problem, both *personalized search engines* and *recommendation strategies* implemented within social media aim to help users in retrieving information which is relevant with respect to their interests. However, providing information that is *credible* as well as topically relevant, is still a difficult and ongoing problem (Viviani and Pasi 2017; Putri et al. 2020). Furthermore, social media platforms tend to emphasize some of the congenital, social, and psychological traits of individuals, which lead them to trust, above all, points of view that are similar to their own, disregarding their reliability. Among these traits, *homophily* and *selective exposure* are rather common (Sasahara et al. 2019). The former refers to the principle that a contact between similar people occurs at a higher rate than among dissimilar people (McPherson et al. 2001); the latter to the tendency of people to seek out information that reinforces their ideas and to reject information that threatens them, according to the *confirmation bias* phenomenon (Bessi 2016).

As a consequence of the above-mentioned technological and psychological aspects, social media users are likely to receive information that mostly confirms their viewpoint and, in worse cases, to be “trapped” in a closed information environment of like-minded people. Such environment can easily become a so-called *echo chamber* (Flaxman et al. 2016), in which people “hear their own voice” (Garimella et al. 2018a). In fact, information variety is quite poor among people who share similar beliefs and opinions. Echo chambers have long been criticized, especially for their ability to generate polarization (Del Vicario et al. 2016), and, as a consequence, to increase the *controversy* among the members of online communities (Kumar et al. 2018).

In the literature, some recent studies have tackled the issue of detecting and analyzing echo chambers on social media by considering, in particular, the topological structure of online communities, i.e., the relationships among their members. In this article, we take into consideration, in the online community modeling phase, also some semantic aspects related to the content that is disseminated among individuals, with particular reference to the *sentiment* that emerges from the content itself and the discussed *topics*. Hence, we apply a *community detection* strategy over distinct topology- and content-aware representations of the online community, and we evaluate and discuss the different levels of controversy and homogeneity of the resulting groups.

As a case study, as particularly relevant in the current period and in general as regards the area related to health, we focus on the posts disseminated in the Twitter microblogging platform related to the onset of the COVID-19 pandemic at the beginning of 2020. Experimental evaluations are carried out on these data to assess the effectiveness of the proposed approach.

## Background and related work

A possible definition of *echo chamber* is the one recently provided in the literature by Bruns (2017):

*An echo chamber comes into being where a group of participants choose to preferentially connect with each other, to the exclusion of outsiders. The more fully formed this network is [...] the more isolated from the introduction of outside views is the group, while the views of its members are able to circulate widely within it*.

With this definition in mind, the psychological and non-psychological causes leading to the formation of echo chambers and, hence, to growing social fragmentation and ideological polarization in our society (Del Vicario et al. 2016), are illustrated in the following section.

### Causes of echo chamber generation

The study about the formation of echo chambers is quite recent. Bessi (2016) addressed the problem from the users’ point of view, with a study aimed at displaying common psychological characteristics among the members of different echo chambers. In particular, his study shows that distinct polarized groups are formed around users sharing similar *personality traits*, selected among the so-called big five: *extraversion*, *emotional stability*, *agreeableness*, *conscientiousness*, and *openness* (Costa and McCrae 1999). The prevalent personality model in an echo chamber corresponds to a *prototype-user* who tends to enjoy interactions with close friends (low extraversion), is emotionally stable (high emotional stability), is suspicious and antagonistic toward others (low agreeableness), engages in antisocial behavior (low conscientiousness), and has unconventional interests (high openness).

Quattrociocchi et al. (2016) and Del Vicario et al. (2016) focused on the study of echo chambers and their evolution in Facebook. In the first work, the authors study specific actions such as *share*, *comment*, or *like* with respect to their meaning in exposing to/appreciating/supporting information in the social media platform. Based on authors’ findings, users are actually highly polarized on Facebook, since they focus on like-minded people’s posts (homophily) and seek out posts that strengthen their ideas (selective exposure). In the second work, the authors show that *two* different echo chambers evolve in a similar way due to the similarity among their members’ behavior; in particular, it seems that users’ polarization depends on their level of *involvement* in the community. The more active users are, the more polarized are the opinions they share.

Sasahara et al. (2019) have also studied the conditions in which echo chambers emerge. The proposed model’s dynamics indicate that the online community rapidly evolves into isolated, homogeneous groups, even with small amount of *influence* (related to the concept of involvement previously discussed), and *unfriending*. Moreover, their study shows that social media debates tend to polarize individuals in exactly *two* opinion groups. They also find empirical evidence that, in many cases, the presence of users with many followers (*hub* nodes) affects the dissemination of the same messages. Furthermore, their study suggests that *triadic closure* connects individuals to friends of their friends, facilitating repeated exposure to the same opinion. Such echoes are a powerful reinforcing mechanism for the adoption of beliefs and behaviors.

Recently, in the Twitter scenario (the same considered in this article), Baumann et al. (2020) have proposed a model introducing the dynamics of radicalization as a reinforcing mechanism driving the evolution to extreme opinions from moderate initial conditions. The outcome of the work illustrates that the transition between a global consensus and emerging radicalized states is mostly governed by *social influence* and by the “controversialness” of the topic discussed.

### Echo chamber detection

Often, a virtual community is formed around a specific *topic* of interest. This is especially true in those social media which are mainly focused on the exchange of textual content and the generation of the so-called *conversational threads* (Aragón et al. 2017). In this scenario, there are specific topics that lend more than others to the formation of echo chambers (e.g., politics, health, religion, etc.) (Baumann et al. 2020), which are usually polarized around *two* main points of view (Sasahara et al. 2019). Here, the concepts of *controversy* (Garimella et al. 2018b) and *homogeneity* (Quattrociocchi et al. 2016; Sasahara et al. 2019) become fundamental for the recognition of echo chambers, interpreted as *polarized* groups of individuals. The higher the controversy between members of different groups and the homogeneity between members of the same group, the higher the probability to be in the presence of echo chambers.

Recent works in the literature whose aim is to identify *binary* polarization (i.e., two strongly polarized groups of individuals) in virtual communities mostly rely on the quantification of controversy/homogeneity after performing *graph partitioning* on the *graph-based representation* of social interactions around a given topic, also known as *conversation graph*.

Therefore, referring in particular to the work of Garimella et al. (2018b), in this paper we present as a possible scenario for the identification of echo chambers the one constituted by the following three phases:

1. *Modeling* the conversation graph (i.e., the graph of interactions among users around a topic of interest);
2. *Partitioning* the graph into two groups by selecting/defining a suitable strategy;
3. *Quantifying* the polarization of the members of the groups obtained in the previous step by assessing controversy/homogeneity.

#### Modeling the conversation graph

Online communities are *complex networks* whose members and relationships are usually represented and analyzed by means of *graph theory* and *Social Network Analysis* (SNA) techniques (Knoke and Yang 2019). From a formal point of view, a *graph* $G=(V,E)$ consists of a pair of sets: a set *V*, which is called the *set of vertices*, i.e., $V = \{v_{1}, \ldots , v_{n} \}$, and a set *E*, which is called the *set of edges*. *E* is a subset of the Cartesian product $V \times V$, i.e., it is a set of pairs $e_j=(v_{k}, v_{l})$, with $j=1,\ldots ,m$, and $v_{k}, v_{l} \in V$.

In general, in the graph-based representation of a virtual community, the vertices represent the members of the community, and the edges possible social interactions between them. Different social media platforms can lead to the generation of many different kinds of relationships among their users (Carminati et al. 2012), e.g., a *friendship* in Facebook, a *followee/follower* relationship in Twitter, a *comment* to someone’s post in Instagram, etc.; this happens also within the same social media platform, e.g., the *followee/follower*, the *retweet*, and the *mention* relationships in Twitter, which will be better detailed in Sect. 3.1.1.

As previously illustrated, recent works investigating the echo chamber phenomenon mainly focus on those social platforms, such as Twitter, and social relationships that allow the construction of conversation graphs. In this context, for example, the work by Garimella et al. (2018b) proposes to model the three conversation graphs built around two controversial (i.e., #Ukraine and #BeefBan) and one non-controversial (i.e., #NationalKissingDay) hashtags, collected on Twitter, via an undirected graph representation where vertices are Twitter users and edges represents both a *retweet* or a *followee*/*follower* relationship between them. Also in the work by Coletto et al. (2017), different graphs are constructed with respect to different controversial and non-controversial topics on Twitter. In this case, for each tweet collected with respect to a topic, the *discussion thread* is reconstructed by performing a crawling operation on the replies to the initial tweet. In this case, a graph relating to the *followee/follower* relationship between two users is constructed, as well as a graph relating to the fact that a tweet is the *reply* to another tweet; hence, by using these two graphs, a further graph is generated, in which an edge between two users represents the *reply* of a user to another user’s tweet. Also in the work by Kumar et al. (2018), the community is modeled through the use of a *reply tree* which involves, given a tweet, all the related interactions.

#### Partitioning the graph

Recent works of the literature addressing the problem of echo chamber detection over a conversation graph have proceeded to use *community detection* algorithms for partitioning the graph into *two* distinct groups, a.k.a. *communities*. As stated in (Papadopoulos et al. 2012):

*Community detection constitutes a significant tool for the analysis of complex networks by enabling the study of mesoscopic structures* [...] *often associated with organizational and functional characteristics of the underlying networks*.

The first literature works that have addressed the problem of community detection in complex networks (regardless of the echo chamber phenomenon) have often focused on methods that analyze only the *topological* structure of the network (Fortunato and Hric 2016). In these approaches, usually the number of intra-group connections compared to the number of extra-group connections is considered in some way.

However, considering only topological aspects is problematic mainly for two reasons, particularly in online communities: (i) the graph-based modeling of the community depends on the type of relationships that are taken into account, as previously illustrated; (ii) the content associated with social interactions is ignored, not allowing to “enrich” the topological representation of the conversation graph with semantic information. As stated by Natarajan et al. (2013), a link between two users increases the chances that the two share common interests, but does not necessarily imply it. Furthermore, two users who do not share a link might still have common interests.

Therefore, for some years now, the works that deal with community detection have been trying to consider some semantic information such as the *sentiment* linked to the exchanged content and *topics* of interest (within the more general topic around which the community is generated) that the community members discuss. This is the case, among others, of the approaches proposed by Pathak et al. (2008), Sachan et al. (2012), Natarajan et al. (2013), Zhang et al. (2015), Sawhney et al. (2017).

As for the community detection algorithms employed in the identification of echo chambers by recent literature approaches, they are mostly topology-based, not taking sufficiently into account specific semantic aspects (Garimella et al. 2018a, b; Dokuka et al. 2018; Cossard et al. 2020), or they are applied separately to topology- and content-based representations of conversation graphs (Yuan and Crooks 2018). In this work, on the contrary, our aim is to consider these aspects together in the graph modeling and partitioning phases.

#### Quantifying polarization

The last echo chamber detection phase consists in quantifying the polarization of the identified partitions, by assessing controversy and/or homogeneity within them. This activity serves to verify the effective presence of echo chambers in the virtual community.

In the work by Coletto et al. (2017), structural aspects of the network are mainly taken into account to assess polarization (e.g., the number of vertices, edges, and degree distribution), as well as propagation information (e.g., the reply tree depth), temporal information (e.g., the time elapsed between one reply and the next one), and local information (e.g., how many replying users are connected by a *followee*/*follower* relationship). Del Vicario et al. (2016) focus mainly on the concept of homogeneity in diffusing specific kinds of contents, while both structural and content aspects to assess controversy and homogeneity are discussed by Sasahara et al. (2019). However, one of the most complete work from this point of view is the one proposed by Garimella et al. (2018b), which illustrates specific metrics for evaluating the level of controversy between user groups, which will be detailed in Sect. 3.3.

## A topology- and content-based approach for echo chamber detection and analysis

The approach proposed in this work and detailed in this section, aimed at detecting and analyzing echo chambers, is framed in the context of the COVID-19 pandemic discussions on the Twitter microblogging platform at the beginning of 2020 (roughly in the first months of its diffusion).

First, the modeling of the conversation graph built on the available COVID-19 data is illustrated, leading to four distinct graph representations based on both topological and semantic aspects; then, the chosen partitioning algorithm and its application to the four above-mentioned representations of the conversation graph are detailed; finally, the measures and other qualitative methods employed to assess the level of controversy and homogeneity with respect to the groups identified by the partitioning algorithm (under the four representations) are presented.

### Modeling the conversation graph

The modeling phase of a conversation graph is strongly dataset-dependent; for this reason, we first introduce the data that were collected and employed in this work before discussing modeling aspects.

#### The considered dataset: COVID-19 on Twitter

The data considered relate to tweets (and connected metadata) downloaded from Twitter in the period January 15, 2020–March 15, 2020, discussing the COVID-19 pandemic, originally collected for evaluating the psychological impact of the virus on people through the analysis of social media content (Crocamo et al. 2020).

The considered period represents more or less the starting phase and the subsequent explosion of the discussion about this virus in the Twitter microblogging platform. At that time, the name COVID-19 had not yet been proposed, so the data was collected by crawling tweets containing the term *coronavirus* in their texts and hashtags. Through this process, approximately 10 million tweets (in four different languages: English, Spanish, Italian, and French) related to around 4 million of unique Twitter accounts were collected.

#### Topological structure of the graph

The Twitter microblogging platform allows to generate short-text messages (up to 280 characters), i.e., *tweets*, which can be classified as:^1^

- *General tweets*: messages posted to Twitter containing text, photos, a GIF, and/or video;
- *Mentions*: tweets containing another account’s Twitter username, preceded by the “@” symbol;
- *Replies*: responses to another person’s tweet;
- *Retweets*: re-postings of a tweet.

In addition, Twitter allows users to *follow* one another’s tweets. In general, therefore, it is possible to build a conversation graph on Twitter data by using information relating to mentions, replies, and retweets, or by considering the followee/follower relationship. Unlike previous works, mainly focused on the latter type of relationship (as illustrated in Sect. 2.2.1), in this work we have privileged the *mention* relationship. In fact, we want to focus on the explicit involvement of the content in the establishment of a social interaction.^2^

Thus, in the modeling of the COVID-19 conversation graph, the $v_i$ vertices represent users in Twitter discussing the COVID-19 virus, while the $e_j$ edges represent mentions among them. In particular, the graph is *undirected* (a graph where all the edges are bidirectional), *simple* (a graph that has neither self-loops nor parallel edges), and *weighted*; in this *topology-based modeling* of the graph, the weight $w_t$ on an edge represents the *number of mentions* between two vertices.

**Fig. 1:**
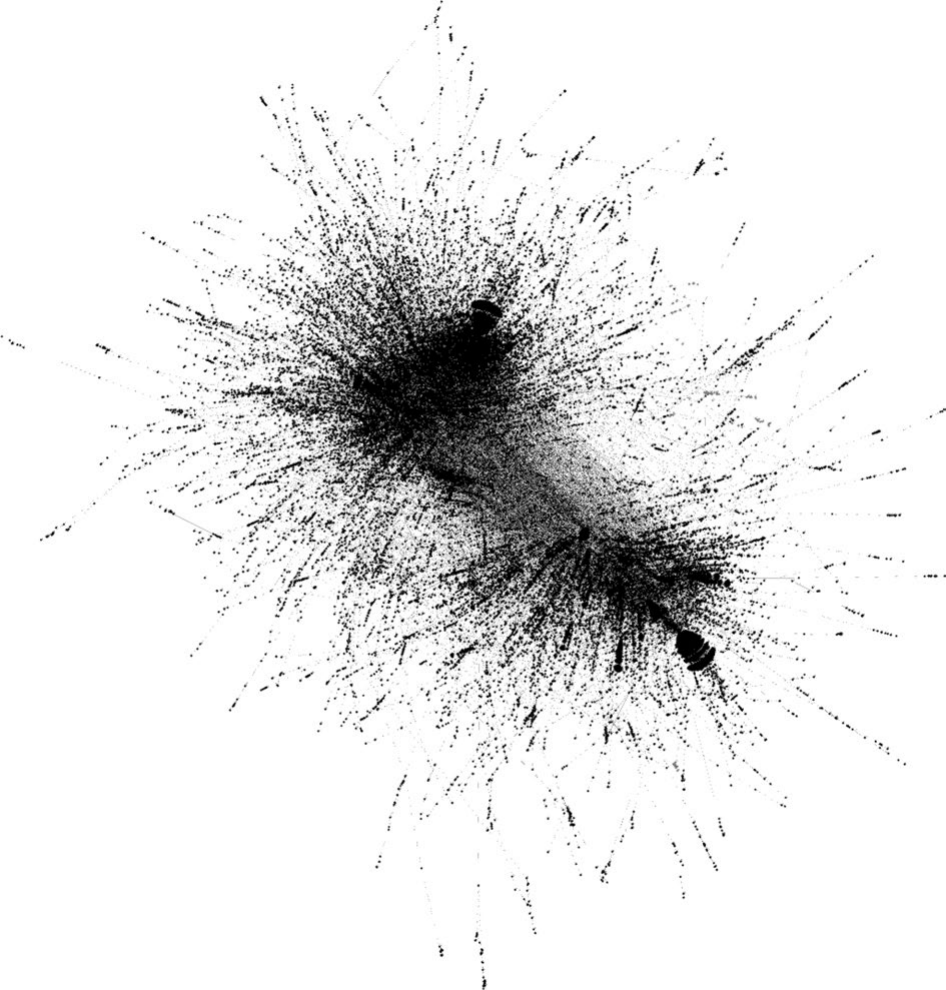
Representation of the COVID-19 conversation graph obtained with ForceAtlas2, a continuous graph layout algorithm for network visualization designed for the Gephi software, which already aims to identify strongly connected groups (Jacomy et al. 2014)

For the construction of the graph, only the tweets in English were considered. This resulted in a number of tweets equal to about seven million four hundred thousand, with about two million six hundred thousand unique users. Among them, a number of around forty thousand users was involved in “mention” relationships. In order to focus on the most significant component of the conversation graph, we have considered only the edges whose weight was greater than or equal to three, for a total number or around seventy-five thousand edges.^3^ A graphical representation of the resulting graph is provided in Fig. 1, obtained using Gephi.^4^

#### Enriching the graph with semantic information

As briefly illustrated in Sect. 2.2.2, in the research area of community detection algorithms, various approaches have tried to take into account distinct semantic aspects related to content. By considering these approaches, in this work we propose to “enrich” the topology-based representation of the conversation graph with this semantic information, acting in particular on the weights of the edges connecting vertices. The idea is to replace the original $w_t$ weights on the edges in the topology-based representation by new weights that are designed to take into account content-related semantic aspects in addition to topological ones. In particular, taking inspiration from (Sawhney et al. 2017), we consider: (i) the *sentiment* of the tweets, and (ii) the *topics* discussed within the tweets (for example, in the context of the general topic of COVID-19, the topics related to negationism and conspiracy theories, to the need of maintaining social distancing, to the usefulness of wearing masks, etc.); furthermore, (iii) we also propose to consider both aspects together in a hybrid fashion. Such three semantic-based modelings are detailed in the following.

*Sentiment-based modeling.* In this modeling, a *sentiment score* to be associated with each user based on their user-generated tweets is first obtained; then, such score is used to compute a new weight on each edge between users already connected in the topology-based modeling of the graph.

Formally, let us consider a vertex *v* and the sentiment scores $x_1, \ldots , x_t$ associated with its *t* tweets computed in a discrete interval of integers $[-\alpha ,+\alpha ]$,^5^ according to a given sentiment analysis technique; a user sentiment score *s*(*v*) associated with *v* is computed as:

1 $$\begin{aligned} s(v) = \frac{1}{t}\cdot \sum _{j=1}^{t} x_{j}. \end{aligned}$$

Once each user has been assigned a sentiment score *s*(*v*), which, according to its formulation, may vary again between the range values $+\alpha$ and $-\alpha$ (included), it is possible to compute a *sentiment similarity score*, denoted as $ss(v_i, v_j)$, between any couple of users $v_i$ and $v_j$, already connected by an edge in the topology-based modeling, as follows:

2 $$\begin{aligned} ss(v_i, v_j) = 2\alpha - |s(v_i) - s(v_j)|. \end{aligned}$$

This way, a value from 0 to $2\alpha$ is attributed to such couples of users, depending on their sentiment similarity; users with a similar sentiment will have a higher sentiment similarity score with respect to users with a “mixed” or an opposite sentiment.

Finally, the sentiment similarity score is employed to compute, between the considered couples of users, a new weight $w_{ss}$ replacing the $w_t$ weight of the topology-based modeling, according to the following equation:

3 $$\begin{aligned} w_{ss}(v_i, v_j)=1+ss(v_i, v_j). \end{aligned}$$

According to this formulation, in the event of a completely discordant sentiment between two users $v_i$ and $v_j$, i.e., $ss(v_i,v_j)=0$, the weight of the edge will be equal to 1, therefore only the topological component will be considered (the presence of an explicit link between users, regardless of the number of mentions between them). Conversely, if they have a similar sentiment, there will be an increase in the weight of the edge that connects them, even if the number of mentions between them is low. The rationale behind this sentiment-based representation is that we do not want to completely ignore the topological component in the community detection phase, but we want to emphasize that similar groups of users should be identified also by considering the similarity among their sentiment.

In this work, the sentiment of the tweets has been assessed by employing VADER (*Valence Aware Dictionary and sEntiment Reasoner*) (Hutto and Gilbert 2014), a *domain-free*, *lexicon-based* model, which is particularly suitable for the social and microblogging context like that of Twitter (Elbagir and Yang 2020). VADER does not require training, and has defined rules for evaluating *emojis*, *slang*, *uppercase words*, and *grade modifiers,* i.e., words such as *very*, *rather*, *fairly*, and *quite* that impact the sentiment intensity by either increasing or decreasing it (e.g., “I am very scared of the pandemic” is more intense than “I am scared of the pandemic”). When a string needs to be evaluated, VADER returns a dictionary of scores in four distinct categories, i.e., (i) *positive*, (ii) *negative*, (iii) *neutral*, and (iv) *compound*. For the first three categories, a value between 0 and 1 is generated, which represents the proportion of text that falls into each category. The *compound score* is the aggregation of the scores associated with the previous three categories, normalized in an interval ranging from $-1$ (*extremely negative*) to $+1$ (*extremely positive*). In this work, it was decided to use such compound metric, which provides a one-dimensional evaluation of sentiment within a text.^6^ Considering the compound score, a text is defined as *neutral* if its value falls within the interval $[-0.05,0.05]$.

To be employed in the sentiment-based modeling described above, it has been necessary to scale the compound scores produced by VADER with respect to the discrete interval $[-\alpha , +\alpha ]$ previously mentioned. In this work, the value of $\alpha$ has been chosen heuristically, based on some preliminary experiments, and its value has been set to 30. Such scaling was based on a simple linear conversion, formally:

4 $$\begin{aligned}  {scaling}(x) = \frac{(x - {oldMin})\cdot {newRange}}{{oldRange}} + {newMin}. \end{aligned}$$

Specifically, scaling(*x*) represents the scaled value of the original compound score *x*; $$ {oldRange} = ( {oldMax} -  {oldMin})$$, where *oldMin* and *oldMax* are the minimum and maximum compound scores from the old distribution; *newRange* $$=$$ $$( {newMax}$$ − $$ {newMin})$$, where *newMin* and *newMax* correspond to $$-\alpha$$ and $$+\alpha$$, respectively.

*Topic-based modeling.* In this graph modeling, to each user *v* in the graph, is associated a *set of topics* denoted as *T*(*v*), i.e., the subset of topics discussed by *v* within the global set of topics, namely *T*, discussed by all users (in our work, extracted from the COVID-19 conversation graph, see Sect. 4.3.2). The topic similarity $ts(v_i,v_j)$ between any couple of users $v_i$ and $v_j$, already connected by an edge in the topology-based representation of the graph, is computed by considering the overlap of their topics as follows. Formally:

5 $$\begin{aligned} ts(v_i, v_j) = |T| - |T(v_i) {\Delta} T(v_j)| \end{aligned}$$

where $\Delta$ denotes the *symmetric difference* between the two sets $T(v_i)$ and $T(v_j)$. In this way, we assign a topic similarity value that is maximal, and equal to |*T*|, for users with a total overlap of topics discussed, and it is minimal (i.e., equal to 0) when $|T(v_i)\cup T(v_j)|=|T|$ and $|T(v_i)\cap T(v_j)|=0$. Such formulation favors the decrease in topic similarity among users as the number of different topics they discuss increases, while it considers equally similar users who discuss exactly the same topics, whether they are more or less numerous.^7^

As in the case of the sentiment-based modeling of the graph, the new weight $w_{ts}$ replacing $w_t$ in the topology-based representation on the edge connecting $v_i$ and $v_j$ is expressed as:

6 $$\begin{aligned} w_{ts}(v_i,v_j) = 1+ts(v_i, v_j). \end{aligned}$$

According to this formulation, in the borderline case in which $ts(v_i, v_j) = 0$, a weight equal to 1 will be assigned to the edge, which indicates the presence of a topological link.

To associate topics with users, an LDA (*Latent Dirichlet Allocation*) model developed and maintained by the MALLET (*MAchine Learning for LanguagE Toolkit*) project by McCallum (2002) has been employed. For each tweet belonging to the users in the conversation graph, a *tokenization* phase has been performed by using the *TweetTokenizer* offered by NLTK (Bird Steven and Klein 2009);^8^ this allows a better definition of *tokens* by considering the specificity of short texts such as tweets. Then, we eliminated all those tokens that appeared in less than 2% and in more than 50% of the documents, considering, at the end, the first 100,000 tokens given their frequency.^9^ After a phase of stop-words removal and stemming, the Gensim library (Rehurek and Sojka 2010) was used to implement the LDA MALLET model. Given the considered vocabulary, the LDA model has been trained on a number of topics ranging from 2 to 30, to identify the best number of topics to fit the model (Sbalchiero and Eder 2020). For each number of topics in the range, the *topic coherence* metric was evaluated to assess the quality of the obtained topics, and the corresponding trained model saved. Specifically, topic coherence has been computed as the $C_V$ measure discussed in (Röder et al. 2015) and implemented in the models.coherencemodel pipeline in the Gensim library.^10^ The quality of the topics was also evaluated by human assessors; such double evaluation of topics (more details regarding these evaluation aspects will be illustrated in Sect. 4.3.2), have been necessary given the fact that a good topic selection phase is strictly related to the correct semantic enrichment of the conversational graph.^11^

*Hybrid modeling.* Having available the similarity values between users with respect to the sentiment and topics discussed in their tweets, it was decided to develop a hybrid modeling of the conversation graph that took both aspects into consideration at the same time. The objective behind this modeling is to “distance” two users who, speaking in general of the same topics, show a different sentiment, and, vice versa, to “approach” users discussing the same topics and having a similar sentiment. Hence, the hybrid modeling has seen the definition of a further new weight $w_h$, which combines the two previously defined similarity measures. Formally:

7 $$\begin{aligned} w_h(v_i, v_j) = 1 + ss(v_i, v_j) + ts(v_i, v_j). \end{aligned}$$

Again, with this simple weight modeling, if $v_i$ and $v_j$ are totally dissimilar from the point of view of the sentiment expressed by their tweets and the topics they treat over the global set of topics (i.e., $ss(v_i,v_j)=0$ and $ts(v_i,v_j) =0$), the $w_h$ weight (which in this graph representation replaces the $w_t$ weight in the topology-based modeling) will be equal to 1, which indicates the presence of a topological link. The value of this weight will increase as sentiment and topic similarity increase.^12^

### Partitioning the graph

On the four representations of the COVID-19 conversation graph illustrated in the previous section: (i) *topology-based*, (ii) *sentiment-based*, (iii) *topic-based*, and (*iv*) *hybrid*, a community detection algorithm was applied. Since our aim was the selection of an algorithm allowing the identification of *two* distinct groups within the online community, in order to focus on the identification of strong speech polarization, we initially considered different solutions. The algorithms that were identified as suitable for the purpose are, among others, the bisection-based algorithm proposed by Kernighan and Lin (1970), the *FluidC* algorithm, recently proposed by Parés et al. (2017), and the METIS algorithm, proposed by Karypis and Kumar (1995).

The first was discarded being in most of the cases unsuitable for most applications to real-world network data, and for efficiency reasons when applied to this kind of (large and dynamic) networks (Newman 2004); the second was discarded as we experienced, on some preliminary experiments, that it can be highly dependent on the initial choice: by setting different *seeds*, aimed at identifying the node from which to start the partitioning task, the final result can change a lot.

**Fig. 2:**
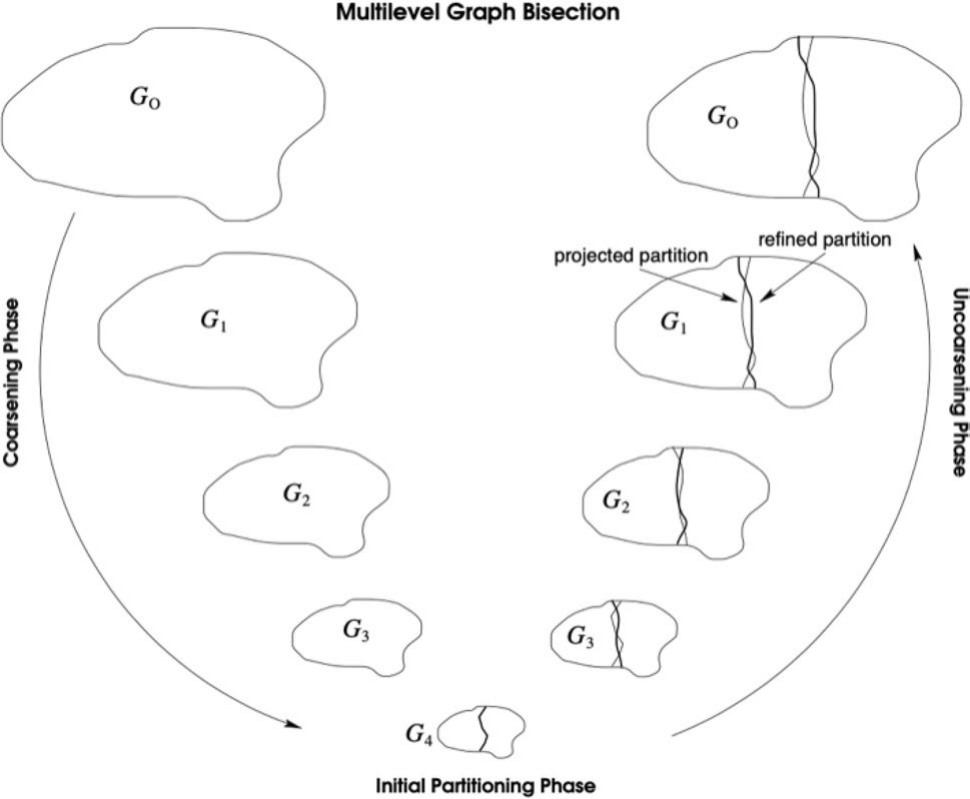
The high-level functioning of the METIS algorithm. Courtesy of George Karypis and Vipin Kumar

The above-mentioned considerations led to the preference of the METIS algorithm (Karypis and Kumar 1995), which allows to obtain two very balanced communities (in METIS, it is possible to define the number of required communities). In addition to this, the algorithm is very efficient with respect to the required computational time, and it has been employed also in the state-of-the-art work proposed by Garimella et al. (2018b). Just to provide a brief overview, METIS is aimed at partitioning undirected graphs, according to the topological characteristics of the network; partitioning is based on a so-called *multilevel graph bisection,* which implies a progressive reduction of the graph, with a subsequent “regrowth” to its original size. Figure 2 graphically illustrates the functioning of the algorithm; for further details, we invite the reader to refer to the original paper (Karypis and Kumar 1995).

In this work, to perform partitioning, we have employed the official METIS 5.x distribution.^13^

### Quantifying polarization

After the conversation graph partitioning phase on the basis of the community detection algorithm applied to the four representations illustrated in Sect. 3.1, it is necessary to proceed with the verification that the obtained partitions can actually be considered as echo chambers. To be able to affirm this, we expect that: (i) there is a high level of *controversy* among the members of different partitions, and (ii) there is a high level of *homogeneity* among the members of the same partition.

There are several measures that, in general, can be adopted to evaluate the goodness of a partitioning; among others, *modularity* and *coverage* (Newman and Girvan 2004; Fortunato 2010). However, these measures have not been proposed in the literature to consider the identification of the echo chamber phenomenon. Therefore, this section briefly describes the measures proposed by Garimella et al. (2018b) to explicitly assess controversy in the echo chamber scenario. In addition to them, we also present a couple of controversy measures defined in this work, as well as some methods based on content analysis to evaluate homogeneity within the identified partitions.

#### Controversy measures

The first two controversy measures considered are based on the concept of *random walk* and *authoritative node*. They aim at capturing how likely it is that a casual user, belonging to a certain community (again, selected at random), can be exposed to the content expressed by an authoritative node of the opposite community. It is assumed that the *degree centrality* of a vertex can be used as an index of its authority, and that a random walk ends when reaching an authoritative node (regardless of community affiliation). The third measure is also based on the random walk concept, but this time considering the number of community changes of a node during a random walk of fixed length. The last measure presented is based on the so-called *boundary connectivity* concept, which measures the degree of connection between so-called internal community and boundary vertices. Details are provided below.

*Random Walk Controversy.* This measure, defined by Garimella et al. (2018b), considers two partitions *X* and *Y* of the graph $G=(V,E)$ (such that $X \cup Y = V$, and $X \cap Y = \emptyset$), and two random walks, one ending in partition *X* and the other ending in partition *Y*. The *Random Walk Controversy* (RWC) measure is defined as the difference of the probabilities of two events: (*i*) both random walks start and end up in the same partition, and (*ii*) both random walks start from a partition and end up in the other one. Formally:

8 $$\begin{aligned} \text {RWC} = P_{XX}P_{YY}-P_{XY}P_{YX} \end{aligned}$$

where $P_{AB}$ with $A, B \in \{X, Y\}$ is a *conditional probability* defined as follows:

9 $$\begin{aligned} P_{AB}=P[\text {starts in A}|\text {ends in B}]. \end{aligned}$$

The value of this metric ranges between 0 and 1. The closer it is to 0, the more likely it is to switch to the other partition (*no controversy*); the closer it is to 1, the more likely it is to stay in the original partition (*presence of controversy*).

*Authoritative Random Walk Controversy.* This measure, proposed in this work, constitutes a slightly modification of the RWC measure; it is denoted as *Authoritative Random Walk Controversy* (ARWC). This name derives from the fact that, if in RWC the selection of starting nodes was completely random between the users of the two different partitions, in ARWC we start only from the nodes defined as authoritative. In a completely similar way to what has already been seen for RWC, the random walk ends once a vertex that is part of the set of authoritative nodes of one or the other partition is reached. In this way, we aim at quantifying how much the authoritative nodes of a partition are exposed to similar individuals, but belonging to the opposite partition. The hypothesis behind the definition of ARWC is that, if an authoritative node is reached by an authoritative node of the other community, it can then more easily influence also the non-authoritative nodes of its own community, thus reducing controversy.

In this work, for both RWC and ARWC, a “restart” mechanism has been implemented: if after a random walk of length equal to the twice *average shortest path* of the graph, no authoritative node is reached, the random selection of another node is performed. The number of nodes randomly selected for both partitions is equivalent, around the 60% of the nodes belonging to each partition; a node is considered as authoritative if its degree is positioned in the top-15% given the community to which it belongs; partitions are selected at random (each with probability 0.5).

*Displacement Random Walk Controversy.* This metric, proposed in this work, and denoted as *Displacement Random Walk Controversy* (DRWC), aims at considering the ratio between the number of steps during a fixed-length random walk leading to a change of community, and the total length of the walk. Formally:

10 $$\begin{aligned} \text {DRWC}= \frac{\sum _{\forall v \in N}\left[ 1-\left( \frac{n(v)_{cc}}{l_{rw}}\right) \right] }{|N|} \end{aligned}$$

where *N* is the set of randomly selected vertices to be considered in computing the measure, $l_{rw}$ is the length of the random walk (the number of edges in the walk), and $n(v)_{cc}$ is the number of steps, in the random walk of *v*, where the node has changed community. The value of this measure ranges in the [0, 1] interval. If a node, during its walk, has never changed community, it means that it is closely connected to its own community, and, therefore, there is controversy between the two communities. If, on the other hand, it crosses the two communities many times, it means that they do not present a high degree of controversy between them. Therefore, higher values of this measure correspond to higher controversy between communities and vice versa.

In this work, for each partition, a number corresponding to about the 60% of the vertices have been randomly selected to compute the metric; the length of the random walk has been defined as twice the *average shortest path* in the graph.

*Boundary Connectivity.* This metric, also employed in (Garimella et al. 2018b) to evaluate controversy between communities, is taken from (Guerra et al. 2013); the measure is based on the concepts of *internal* and *boundary vertices*. Given a graph *G*, let us consider $u \in X$ as a vertex in partition *X*; *u* belongs to the *boundary* of *X* if and only if it is connected to at least one vertex in partition *Y* and to at least one vertex in partition *X* that is not connected to any vertex in partition *Y*. The set of boundary vertices is therefore defined as $B=B_X \cup B_Y$; conversely, the set $I_X = X- B_X$ is the set of internal vertices of partition *X* (in a completely similar way we define the set $I_Y$ for partition *Y*). The set of internal vertices is therefore defined as the set $I = I_X \cup I_Y$. If the two partitions would constitute echo chambers, the whole *B* should be made up of vertices that are more strongly connected with the elements of *I* rather than with elements of *B*.

The following equation, representing *Boundary Connectivity* (BC), formalizes the concept just expressed:

11 $$\begin{aligned} \text {BC}=\frac{1}{|B|}\sum _{u\in B}\left[ \frac{d_{i}(u)}{d_{b}(u)+d_{i}(u)}-0.5\right] \end{aligned}$$

where $d_{i}(u)$ is the number of edges between the vertex *u* and the elements of the set *I*, and $d_{b}(u)$ is the number of edges between the vertex *u* and the elements of the set *B*. BC lies in the range $[-0.5,0.5]$; a BC value below 0 indicates lack of polarization; conversely, a BC value greater than zero indicates that, on average, nodes on the boundary tend to connect to internal nodes rather than to nodes from the other group, indicating that controversy is likely to be present.

#### Assessing homogeneity

In order to assess the level of *homogeneity* of the identified communities, it was decided to take into consideration aspects related to both the *sentiment* of the identified communities, and the *topics* discussed within the communities themselves. In this way it is possible to consider qualitative, “human-based” assessments, aimed at further validating the result of the community detection process in the echo chamber scenario.

Since this is an almost qualitative evaluation, no specific homogeneity measures are presented in this section. The reader is invited to refer to Sect. 4.3 for more details on the evaluation process of this aspect.

## Evaluations

In this section, we present the results of different experimental evaluations connected to distinct aspects of the approach proposed to detect echo chambers on the COVID-19 conversation graph. First, we present some statistics related to the community detection task performed on the four graph representations defined in Sect. 3.1; secondly, we illustrate the results of the quantification of the controversy among partitions based on the controversy measures illustrated in Sect. 3.3.1; finally, we present some results related to the qualitative analysis of the sentiment and the topics of tweets within the identified partitions, as briefly introduced in Sect. 3.3.2.

### Community detection results

**Fig. 3:**
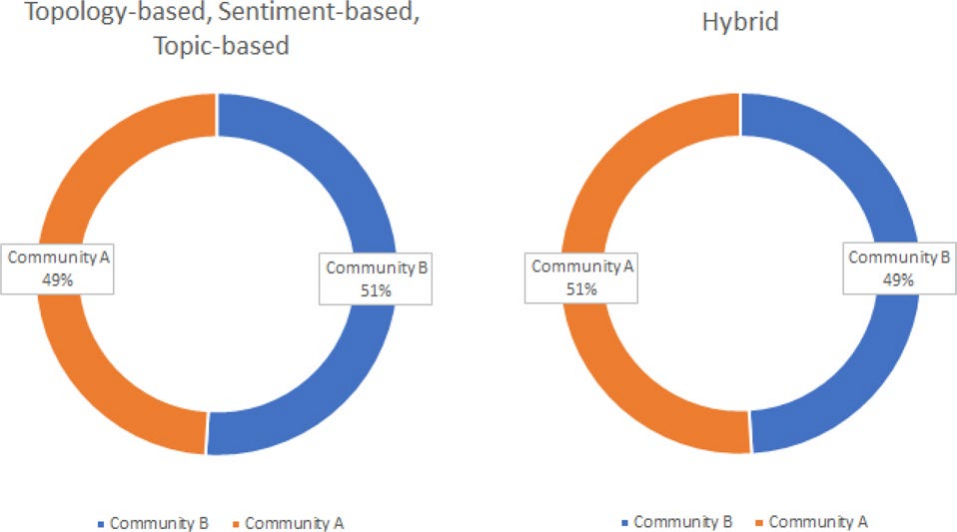
Percentages of the members of the COVID-19 conversation graph partitioned in the two communities A and B based on the four different graph modelings

The results in this section concern a quantitative analysis of the partitioning of the members of the COVID-19 conversation graph by employing METIS applied to the (i) *topology-based* (TP), (ii) *sentiment-based* (SB), (iii) *topic-based* (CB), and (iv) *hybrid* (H) modelings of the graph. The partitioning of members belonging to the two identified communities, namely Community A and Community B, is graphically illustrated in Fig. 3.

From the figure, it can be observed that, regardless of the graph representation employed, the communities obtained through the use of METIS are rather well balanced, even if the hybrid modeling of the graph leads to a slightly different behavior than the other three representations. This can be better explained by considering Table 1, which shows the precise number of members belonging to the two communities, and the number of members that “changed” community with respect to the topology-based representation when considering the other three representations. As it emerges from the table, the number of community changes is extremely high in the case of the hybrid modeling of the graph, leading to the situation illustrated in Fig. 3.

**Table 1:** Number of members belonging to the two identified communities A and B, and community “changes” under the four different graph representations

Type	$|V_\text {A}|$	$|V_\text {B}|$	Changes
TB	19,096	20,240	0
SB	19,163	20,173	6297
CB	19,094	20,242	6920
H	20,240	19,096	32,226

This behavior is probably related to the way in which the weights in the hybrid representation of the conversational graph are computed, via Eq. (7). Such a formalization, where the sentiment and the topic similarity scores are simply summed up, probably leads to a total overturning of the communities distribution. This aspect will have to be statistically analyzed in further research, as discussed later in Sect. 5.

### Controversy results

The results presented in this section illustrate the levels of controversy between the two communities identified by the METIS algorithm with respect to the four different representations of the conversation graph. In particular, Table 2 summarizes the controversy scores obtained by employing both “classic” measures to evaluate the goodness of a partitioning, that is *modularity* (Mod.) and *coverage* (Cov.),^14^ and those measures that have been proposed and defined in the context of the controversy evaluation, that is *Random Walk Controversy* (RWC), *Authoritative Random Walk Controversy* (ARWC), *Displacement Random Walk Controversy* (DRWC), and *Boundary Connectivity* (BC).

**Table 2:** Results of the measures considered to evaluate the controversy between the communities identified by the community detection algorithm on the four representations of the conversation graph

Type	Mod.	Cov.	RWC	ARWC	DRWC	BC
TB	0.4348	0.9351	0.9495	0.8454	*0.9771*	0.1704
SB	**0.4403**	**0.9534**	0.9535	**0.8740**	***0.9807***	**0.1813**
CB	0.4396	0.9403	0.9521	0.8656	*0.9791*	0.1800
H	0.4322	0.9224	**0.9548**	0.8635	*0.9805*	0.1792

From the table, it emerges that, probably because not targeted at the problem at hand, *modularity* does not capture so clearly echo chamber aspects as almost all other measures defined for this purpose seem to do. In particular, it can be seen that the measure that allows to obtain the highest controversy scores between communities is the one based on the count of community changes during the random walk, i.e., DRWC, as indicated in italic in the table, while the least effective one is the one based on boundary connectivity (BC). Furthermore, with respect to the representations of the conversation graphs, it can be observed that the representation that seems to capture the highest values of controversy among the identified communities is that based on sentiment, almost under every controversy measure. This is indicated in bold in the table.

The above-mentioned aspects can be considered as promising in the context of echo chamber detection, even if some of them need to be further investigated, especially those related to the effectiveness of the measures employed, as discussed later in Sect. 5.

### Homogeneity results

This section is dedicated to the presentation of some qualitative analyzes that have been carried out on the two communities obtained by METIS, in particular as regards the analysis of the sentiment and the covered topics. This allows to get an idea of how homogeneous the members of each community are within them.

#### Sentiment analysis results

**Fig. 4:**
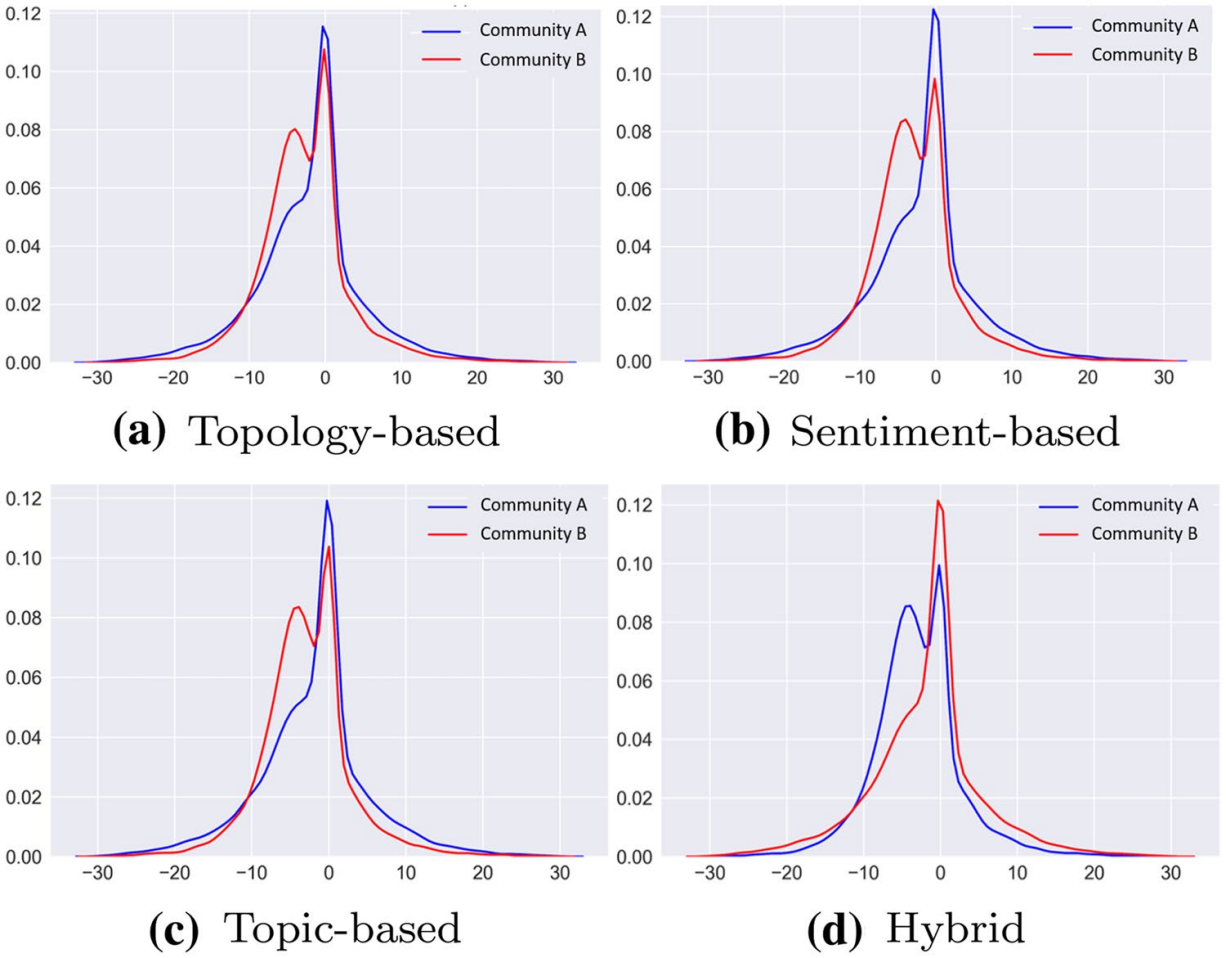
Intra-community sentiment distribution given the different representations used. On the *x*-axis the sentiment scores, while on the *y*-axis their probability

The first qualitative results concern the analysis of the sentiment linked to the tweets of users belonging to the two different communities, in relation to the four different representations of the conversation graph taken into consideration. In Fig. 4, it is possible to visually appreciate the distribution of sentiment within the distinct Communities A and B.

First of all, it is possible to notice, in Fig. 4b, that the sentiment-based modeling “smoothes” the peak of Community B users with neutral sentiment in an important way; we can therefore assume that, given the number of changes made with respect to the topology-based modeling of the graph, these users have been moved to Community A, which in fact sees its peak on neutral sentiment rise. Analyzing the graph in Fig. 4c, we can observe that the flattening of the peak of users with neutral sentiment from Community B is, instead, slightly lost in the topic-based modeling. Finally, the hybrid representation at the basis of Fig. 4d, produces a result that seems almost a reversal of the situation obtained with the approach based on sentiment; remember that the number of users who, given this graph representation, has seen a change of community with respect to the topology-based one, is equal to 32,226, therefore about 82% of the vertices in the graph (see Table 1).

Also this aspect deserves further investigation, as discussed later, again, in Sect. 5.

#### Topic modeling results

**Fig. 5:**
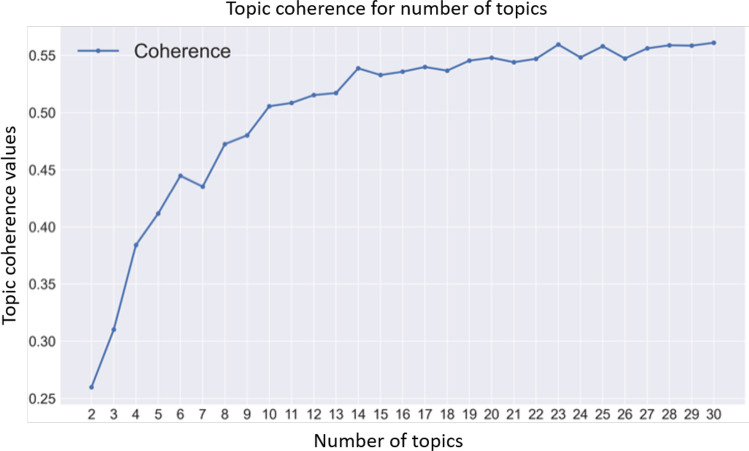
Topic coherence scores obtained with respect to different numbers of topics considered

This section provides an analysis of the topics that have been extracted by means of the topic modeling task presented in Sect. 3.1.3. As previously mentioned, we had to select the LDA model that best performed with respect to the number of topics to be extracted. The optimal results have been obtained with the choice of considering 25 topics. In fact, with this number of topics, we obtain one of the highest *topic coherence* scores, as shown in Fig. 5.

The choice of 25 topics was also confirmed by an analysis carried out by human assessors, based on which it seemed that this number produced the best results in recognizing significant keywords related to the topics considered. Hence, in Table 3 we report, for each topic defined, the most probable, i.e., the top-10, (stemmed) *keywords* that appear in it. As it can be observed from the table, the topics defined, in principle, tend to be characterized by keywords clearly related to specific COVID-19 events, people, lifestyles, etc.: topic 1 seems to talk about the history of the doctor who first tried to alert the authorities about the presence of a new virus; topics 4 and 15 seem to encompass predictions and fears about the impact of the virus on the economy; topic 6 deals with the possible ways of treating this disease; topic 13 concerns Italy and the preventive measures adopted (e.g., lockdown, closure of schools and universities); topic 19 is about the cruise ship case Diamond Princess; topic 21 seems to speak of new hygiene habits; topic 22 has political connotations.

**Table 3:** Top-10 keywords associated with each topic

ID	Top-10 keywords
1	*viru, spread, corona, stop, world, break, news, deadli, continu, friday*
2	*peopl, infect, chines, die, doctor, warn, kill, million, dead, year*
3	*time, inform, read, post, chang, share, import, data, good, prepar*
4	*market, fear, stock, global, point, price, drop, fall, week, worri*
5	*china, outbreak, govern, countri, epidem, control, fight, india, prevent, measur*
6	*vaccin, expert, question, develop, start, month, work, cure, drug, treatment*
7	*health, emerg, public, world, declar, threat, minist, global, nation, intern*
8	*diseas, human, caus, sar, transmiss, scientist, studi, origin, sourc, expert*
9	*iran, home, medic, work, risk, care, stay, worker, sick, famili*
10	*test, patient, posit, hospit, symptom, isol, day, result, contact, neg*
11	*scare, amid, concern, cancel, plan, fear, event, year, grow, major*
12	*death, china, report, toll, number, rise, increas, infect, hubei, provinc*
13	*itali, close, school, region, student, lockdown, countri, italian, univers, shut*
14	*thing, good, happen, money, hope, wait, life, save, love, feel*
15	*impact, economi, global, industri, econom, bank, demand, expect, compani, product*
16	*travel, south, flight, korea, countri, singapor, airlin, australia, airport, restrict*
17	*case, confirm, report, total, recov, bring, number, germani, suspect, today*
18	*state, offici, washington, announc, counti, hong, kong, person, york, health*
19	*quarantin, ship, cruis, japan, passeng, american, princess, evacu, diamond, california*
20	*china, wuhan, epicent, victim, video, citi, citizen, insid, show, beij*
21	*mask, protect, face, hand, prevent, cough, disinfect, food, wash, wear*
22	*trump, respons, presid, blame, penc, democrat, administr, lie, hoax, american*
23	*covid, ncov, pandem, asia, sarscov, canada, wuhanviru, coronavirususa, wuhanpneumonia, wuhan*
24	*updat, news, press, latest, watch, today, follow, outbreak, confer, panic*
25	*polit, polit, problem, claim, real, make, fact, believ, wors, danger*

From the analysis of the topics discussed within the two different communities, under the four graph representations, no significant differences were found with respect to the number of specific topics dealt with more by one or the other community. Most likely what changes is the feeling linked to the discussion with respect to individual topics. Therefore, to get an idea of whether there are substantial differences with respect to specific themes, we have made a further qualitative assessment, described in the next section.

#### Wordclouds

**Fig. 6:**
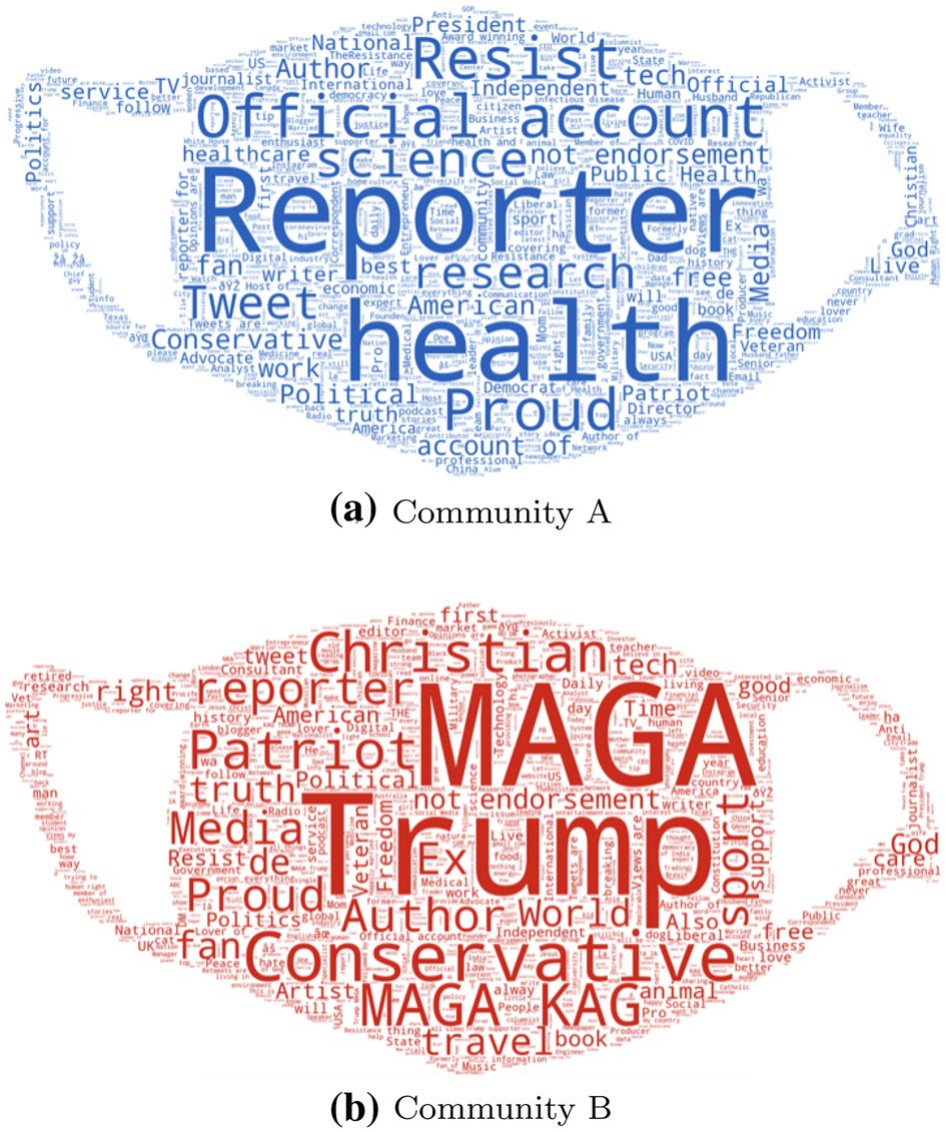
Wordclouds obtained given the description of Twitter accounts according to their community of belonging (under the *sentiment-based* representation)

In this qualitative analysis, we have considered the descriptions associated with the Twitter accounts of the users belonging to the two communities. For each account description, the most important *keywords* employed by the user to define their beliefs and thoughts have been extracted, and illustrated by means of *wordclouds*. Figure 6 presents these wordclouds related to members of Community A and Community B under the *sentiment-based* graph representation, i.e., the most effective in capturing controversy (Sect. 4.2).

Figure 6a, referring to Community A, appears to be endowed with words recalling the scientific and the information community; words like *research*, *health*, *science*, *healthcare*, *public health*, and *journalist*, can actually be referred to this area and, more generally, to an informed discussion on the COVID-19. On the other hand, Fig. 6b, referring to Community B, contains words that clearly recall a political orientation aimed at supporting the candidate Donald John Trump in the 2020 US presidential election; acronyms like MAGA (*Make America Great Again*) or KAG (*Keep America Great*), recall the candidate’s election spots. Furthermore, words like *conservative*, *patriot*, *christian*, and *God* recall certain core values on which the Republican Party in the United States is based.

Given these wordclouds, and taking into consideration the graph illustrated in Fig. 4b, about the distribution of average user sentiment given the sentiment-based representation of the COVID-19 conversation graph, it can be concluded that Community B, which seems more politically oriented, presents a much more negative sentiment than Community A, which seems more scientifically oriented.

#### Verified VS unverified accounts

Another interesting qualitative evaluation related to homogeneity, concerns the possible contrast regarding the reliability of the information exchanged on the conversation graph. One possibility to evaluate such reliability concerns the identification of the number of users, for each community, with a *verified account*. In Twitter, in fact, a *blue badge* associated with an account lets people know that the account is actually of public interest and authentic.^15^

**Fig. 7:**
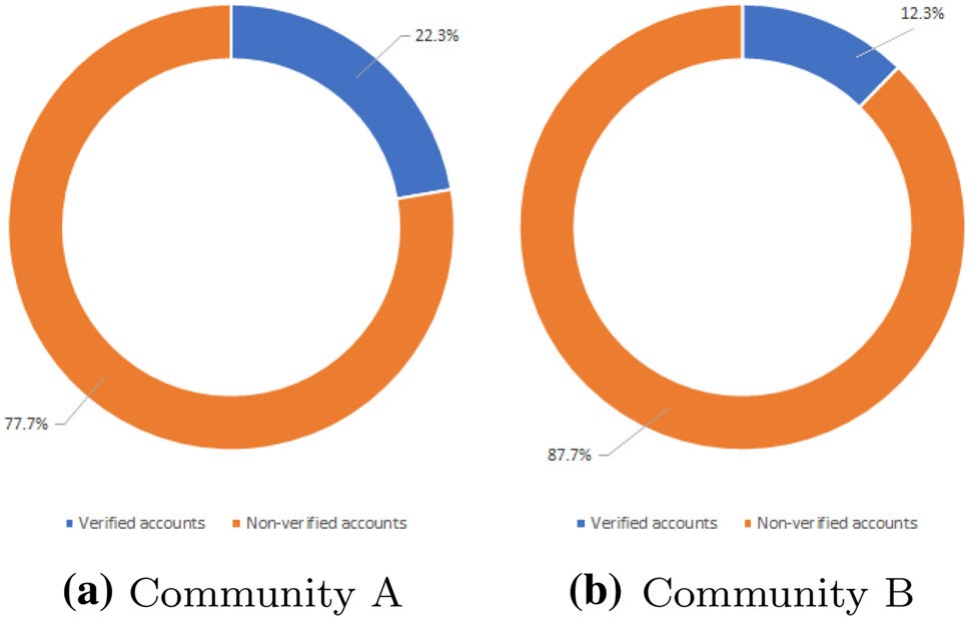
Percentage of blue verified badge accounts in Twitter for each community (under the *sentiment-based* representation)

As shown in Fig. 7, the number of verified accounts for the Community A fluctuates between 22 and 23% of the total number of accounts. Community B, on the other hand, has only 12% of verified accounts.

**Fig. 8:**
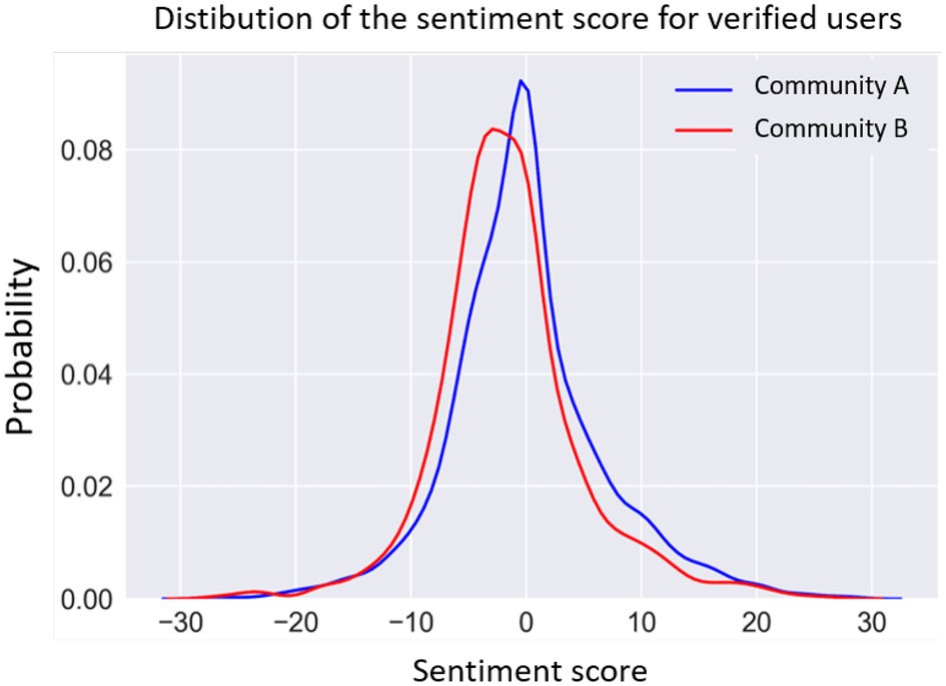
Distribution of sentiment scores for verified users in both communities (under the *sentiment-based* representation)

Another qualitative analysis was conducted based on the *average sentiment*, for each partition, of users with the blue badge. As it can be seen from Fig. 8, verified users of Community B have a more negative average sentiment than verified users of Community A, who instead have a more neutral sentiment.

With respect to Fig. 4b, we notice that the neutral peak of the curve disappears relating to Community B, while Community A maintains more or less the same neutral trend. This is most likely due to the fact that the verified accounts belonging to the two communities pursue different goals, as also previously noted in Fig. 6: a more informative and scientific purpose of Community A; a more support and propaganda purpose of Community B. The absence of unverified accounts in such an assessment which leads to Fig. 8, therefore allows a better identification of these purposes.

## Conclusions and further research

In this article, we have addressed the problem of detecting and analyzing echo chambers in social media, in particular in the Twitter microblogging platform, relating to the first phase of the spread of the 2019 Coronavirus disease, between January and March 2020.

Specifically, the conversation graph built around the tweets discussing the COVID-19 pandemic has been modeled, by considering both topology- and content-based aspects. In this way, we have obtained four different representations of the graph, one based only on explicit relationships (i.e., mentions) between tweets, and the others considering also the sentiment of tweets, the topics discussed, and both aspects together. Then, on these conversation graph modelings, we have applied a well-known community detection algorithm (i.e., METIS) to partition the graph in two distinct communities. To verify that the obtain communities were indeed echo chambers, they have been evaluated: (*i*) with respect to some classic graph partitioning measures (i.e., modularity and coverage), (*ii*) to some controversy measures proposed both in the literature and in this article, and (*iii*) with respect to their level of homogeneity, by means of a qualitative analysis. The results obtained in relation to these evaluations have made it possible to highlight, first of all, that taking into account semantic aspects in modeling the conversation graph is certainly useful for echo chamber detection with respect to considering only topological aspects. In this sense, it will certainly be interesting to also consider possible modeling of the graph that add or remove links on the basis of considerations exclusively related to the exchanged contents.

However, while it has become fairly clear that the addition of semantic information may have a contribution with respect to the problem under consideration, further investigation is needed to clarify whether those identified are strongly or rather weakly formulated echo chambers. In fact, although most of the controversy measures have confirmed the presence of echo chambers in a pretty strong way (and this has also been confirmed by the qualitative analyzes, at least for the sentiment-based modeling), modularity and boundary connectivity have not confirmed this outcome so clearly. It is true that in the proposed approach we considered a binary partitioning of the conversational graph and this may have affected modularity, a measure that is not targeted on the echo chamber problem; however, the results obtained through its use and those obtained through boundary connectivity should be further investigated, to assess the effectiveness of such measures with respect to the considered problem.

Related to the above-mentioned issue, another interesting aspect to be tackled concerns the definition of new measures for the assessment of controversy. Some of the state-of-the-art measures are based on the concept of random walk, but the limit of this modeling is to be found in the fact that each edge has the same probability of being followed along the walk. In reality, this does not happen; users are encouraged to choose certain paths with respect to others based on psychological and technological reasons.

Additional investigations could be related to graph modeling and community detection algorithms. In this work, we focused on the use of mentions to build the topology-based graph; taking into consideration different types of relationships is certainly an aspect to be further deepened. In addition to this, the virtual community has been modeled as an undirected graph, as performed in other works in the literature. This is a commonly adopted solution because effective community detection algorithms are mostly developed for this type of representation. It would be worthy of investigation the study of the problem even in the presence of an oriented representation of the graph. This could be useful not only in the case of binary partitioning of the graph, but also with respect to the identification of a higher number of sub-communities within the conversation graph.

As for taking semantic aspects into consideration, in this work we have shown how the use of sentiment linked to tweets can be particularly effective in the study of echo chamber detection. Regarding the results obtained considering topic-based and hybrid representations, even if they too have led to obtain better results than those obtained with the topology-based representation alone, however, new studies should be performed from different perspectives.

As for the representation that takes into account topics, other tokenization rules and topic configurations should be investigated to verify that these choices do not affect the detection of echo chambers. Regarding the hybrid representation, its current formulation could lead to borderline situations where one aspect (sentiment or topic) dominates the other; furthermore, it has been observed that it led to a near reversal of community members. The above-mentioned issues, and the possible interaction between them, need an in-depth study and statistical analyzes to verify if the identified echo chambers are not affected by this formulation.

Connected to the above-mentioned aspect, there is the need of studying in more detail the relationship between the topics discussed and the sentiment linked to these topics, an aspect that in this work has been only partially considered.

## Data Availability

Data are available upon request.

## Footnotes

1 https://help.twitter.com/en/using-twitter/types-of-tweets.

2 This choice has been undertaken to better concentrate on a single semantic aspect of the problem and better identify the echo chamber phenomenon. However, this choice does not limit the proposed approach to the use of other types of relations (even together), as discussed in Sect. 5.

3 The size of this graph is comparable to those built on several datasets used for the study of the controversy in conversation graphs, as illustrated in (Garimella et al. 2018b).

4 https://gephi.org/.

5 Considering a discrete interval of values is functional to the fact that in the topology-based undirected and weighted representation of the conversation graph, the weights on the edges are integer values representing the number of mentions among users’ tweets, and to the usage of the community detection algorithm that will be described in the next section.

6 https://github.com/cjhutto/vaderSentiment.

7 Different solutions based on increasing similarity as common topics increase can be investigated in future research.

8 https://www.nltk.org/api/nltk.tokenize.html.

9 According to Luhn (1958), terms with medium frequencies are more important concerning their significance than terms that have low or high frequencies; according to Salton et al. (1983), it is necessary to mitigate the effect of such terms to be significant in relevance determination.

10 https://radimrehurek.com/gensim/models/coherencemodel.html.

11 However, it may be useful to consider and test more than one configuration of topics in the future refinement of the approach. The same can be said for the token selection phase.

12 Given its simple formulation, it may be that there are borderline cases where one of the two components (i.e., either the sentiment or the topic), could dominate the other. This possible behavior of the modeling, and the need to further investigate it, will be discussed in Sects. 4.1 and 5.

13 METIS on the Karypis Lab Website: http://glaros.dtc.umn.edu/gkhome/metis/metis/download.

14 Computed as the *Louvain modularity* detailed in (Newman and Girvan 2004), and as detailed in (Fortunato 2010) for *coverage*.

15 https://help.twitter.com/en/managing-your-account/twitter-verified-accounts

## Declarations

### Code availability

The code is publicly available at the following URL—https://github.com/ikr3-lab/echochambers/.

### Gender neutrality in language

In this article, the *singular they* form has been used to make the article gender-neutral, according to the guidelines provided at the following URL—https://apastyle.apa.org/style-grammar-guidelines/grammar/singular-they.
